# Risk factors of severe hypoglycemia among patients with type 2 diabetes mellitus in outpatient clinic of tertiary hospital in Indonesia

**DOI:** 10.1038/s41598-023-43459-2

**Published:** 2023-09-27

**Authors:** Em Yunir, Antonius R. A. Nugraha, Martha Rosana, Juferdy Kurniawan, Eni Iswati, Angela Sarumpaet, Tri Juli Edi Tarigan, Dicky L. Tahapary

**Affiliations:** 1 Division of Endocrinology, Metabolism, and Diabetes, Department of Internal Medicine, Dr. Cipto Mangunkusumo National General Hospital, Faculty of Medicine Universitas Indonesia, Jakarta, Indonesia; 2 Metabolic Disorder, Cardiovascular and Aging Cluster, Indonesian Medical Education and Research Institute, Faculty of Medicine Universitas Indonesia, Jakarta, Indonesia; 3 Department of Internal Medicine, Dr. Cipto Mangunkusumo National General Hospital, Faculty of Medicine Universitas Indonesia, Jakarta, Indonesia; 4 Clinical Epidemiological Unit, Department of Internal Medicine, Dr. Cipto Mangunkusumo National General Hospital, Jakarta, Indonesia; 5 Division of Hepatobiliary, Department of Internal Medicine, Dr. Cipto Mangunkusumo National General Hospital, Faculty of Medicine Universitas Indonesia, Jakarta, Indonesia

**Keywords:** Endocrinology, Risk factors

## Abstract

This study aimed to describe risk factors of severe hypoglycemia in type 2 diabetes mellitus (T2DM) patients in a tertiary care hospital in Indonesia. This study was a retrospective cohort study in the Endocrinology Outpatient Clinic of Dr. Cipto Mangunkusumo National General Hospital, Jakarta, Indonesia. All subjects more than 18 years old who had been visiting the clinic for at least a year were included. Subjects were interviewed whether they had any severe hypoglycemia events within the past year, while data on risk factor variables of severe hypoglycemia was taken from medical records one year before data collection. We recruited 291 subjects, among whom 25.4% suffered at least one episode of severe hypoglycemia within one year. History of severe hypoglycemia (OR 5.864, p ≤ 0.001), eGFR less than 60 mL/min/1.73m^2^ (OR 1.976, p = 0.028), and insulin use (OR 2.257, p = 0.021) were associated with increased risk of severe hypoglycemia. In conclusion, history of previous severe hypoglycemia, eGFR less than 60 mL/min/1.73m^2^, and insulin use were associated with severe hypoglycemia.

## Introduction

Hypoglycemia is one of the most common acute complications of T2DM^1,2^. Neuroglycopenic manifestations of hypoglycemia range from mild to lethal symptoms that can compromise patient safety and are potentially deadly^3^. Severe hypoglycemia is defined as an event of hypoglycemia associated with severe cognitive impairment requiring external assistance for recovery, regardless of the plasma glucose concentration^4^. A prospective cohort study by Rudijanto et al.^5^ showed that 99.4% of type-2 diabetes patients had one episode of hypoglycemia. Moreover, the incidence rate of any hypoglycemia was 25.7 events per patient-year for T2DM patients in Indonesia^5^. Severe hypoglycemia was associated with increased cardio-cerebrovascular events, fear of hypoglycemia, higher risk of dementia, a longer length of stay (LOS), and higher medical expenses^6,7^. Therefore, the identification of clinical risk factors for severe hypoglycemia is of paramount importance.

Studies in various countries have reported several risk factors that are significantly associated with severe hypoglycemia, including age, level of education, knowledge of hypoglycemia, HbA1c levels, duration of T2DM, chronic kidney disease (CKD), chronic liver disease (CLD), history of previous severe hypoglycemia, self-monitoring of blood glucose (SMBG), and types of hypoglycemic agents^2,8–13^. However, those studies have reported inconsistent results. This might be due to the differences in the essential characteristics of the T2DM patients, study settings, and resources available.

According to the International Diabetes Federation (IDF), there were 19.5 million people with diabetes in Indonesia in 2021, and it predicted raised up to 28.6 million people with diabetes in 2045 so this study is important for health workers, particularly in Indonesia^14^. Despite many diabetes patients in Indonesia, there is a scarcity of data on severe hypoglycemia. If anything, two studies in Indonesia on severe hypoglycemia, such as the International Operations Hypoglycemia Assessment Tool (IO HAT) study^5^ and a study from Jayanti et al.^15^ were conducted in specific T2DM populations (in insulin-treated T2DM and hospitalized T2DM population, respectively). Our study aims to describe the prevalence and risk factors of severe hypoglycemia among T2DM patients in an outpatient setting of a tertiary care hospital as well as a national referral hospital in Indonesia. The majority of patients at our hospital are referred from primary or secondary health care, in which patients have to return to primary or secondary health care after three months at our hospital so the data is important as input for evaluating patients in primary or secondary health care. Additionally, the study location acts as a teaching institution for a general practitioner program, 31 specialist programs, and 6 subspecialist programs^16^.

## Methods

This study was a retrospective cohort study conducted at the Endocrinology Clinic, Dr. Cipto Mangunkusumo National General Hospital, a tertiary-care hospital located in Jakarta, the capital city of Indonesia. The clinic treats patients with various complications. Based on medical records, there were 4129 subjects with diabetes in the Endocrinology Clinic in 2022. A consecutive recruitment method was performed from October 2019 to January 2020. The inclusion criteria were T2DM patients, aged more than 18 years, who had regularly visited the clinic for at least one year. The exclusion criteria were pregnancy, psychiatric disorders, and insufficient clinical information recorded. This study was approved by Ethical Committee of Faculty of Medicine, Universitas Indonesia (KET-984/UN2.F1/ETIK/PPM.00.02/2019). Informed consent was obtained from all subjects. All methods were carried out according with relevant guidelines and regulations.

The primary outcome of this study was any event(s) of severe hypoglycemia within the past year, which was asked to the subject during the subjects’ visit. We used the 2018 American Diabetes Association (ADA) definition of severe hypoglycemia, which was an event of hypoglycemia, associated with severe cognitive impairment requiring external assistance for recovery, with or without plasma glucose concentration^17^. The risk factors for severe hypoglycemia included in this study were age, level of education, subjects understanding of hypoglycemia symptoms, HbA1c levels, duration of T2DM, chronic kidney disease (CKD), decompensated chronic liver disease (CLD), history of previous severe hypoglycemia, self-monitoring of blood glucose (SMBG) application, sulfonylurea (SU) use, and insulin use. Data regarding those risk factors were taken from the subjects’ medical records one year before data collection, except for subjects’ understanding of hypoglycemia.

Subjects’ understanding of hypoglycemia was defined as subjects’ ability to mention at least three neuroglycopenic symptoms of hypoglycemia^18^. Chronic kidney disease (CKD) was defined as having impaired renal function for at least three months, assessed based on estimated glomerular filtration rate (e-GFR)^18^. Decompensated CLD was defined as having Child–Pugh Score of more than 10 (C)^19^. Self-monitoring of blood glucose (SMBG) application was defined as well-performed if the frequency of blood glucose monitoring in a week was by the recommendation from the Indonesian Society of Endocrinology (PERKENI), which vary according to subjects' T2DM treatment regimen^20^.

Statistical analysis was conducted using SPSS Statistics for Windows, Version 20.0. Missing risk factors data were filled in using multiple imputation techniques. We presented continuous variables with mean and standard deviations (SD) or median and interquartile range (IQR), while nominal variables with counts and percentages. The normality test was assessed using the Kolmogorov Smirnov. Bivariate analysis was performed using chi-square test. Using a multiple logistic regression test, all variables in bivariate analysis with a p value less than 0.25 were included in multivariate analysis. The estimation of association was presented as odds ratio (OR). The risk factor model was developed by calculating the coefficient formula divided by the standard error for the related variable.

## Results

**Figure 1:**
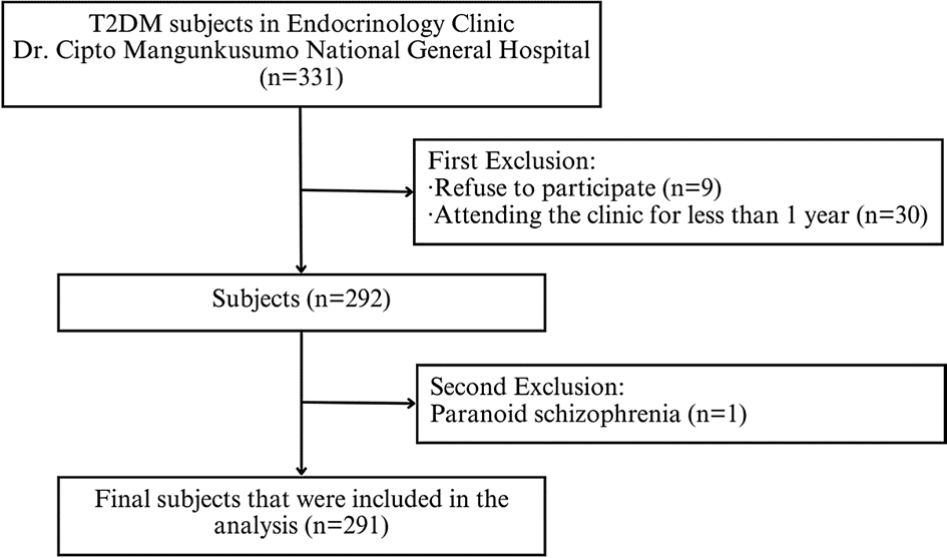
Flow diagram summarizing subject recruitment process.

We recruited 331 subjects, of whom 40 were excluded for various reasons (Fig. 1), resulting in 291 subjects being included in the final analysis. The characteristics of these subjects are presented in Table 1. The median age of our subjects was 61 (54–66) years, and most subjects were female (62.9%). Most of the subjects received insulin therapy (60.8%). In our study, 20.6% of subjects had a history of previous events of severe hypoglycemia. The median duration of T2DM was 12 (5–19) years, with median HbA1c was 7.5 (6.5–8.7). Meanwhile, the majority of the subjects had CKD stage II (33.7%).

**Table 1:** Characteristics of subjects.

Variable	Value
	N = 291
Age (years), median (IQR)	61 (54–66)
Female, n (%)	183 (62.9)
Education level less than 9 years, n (%)	77 (26.6)
Subjects’ understanding of hypoglycemia symptoms, n (%)	144 (49.5)
History of severe hypoglycemia, n (%)	60 (20.6)
HbA1c (%), median (IQR)	7.5 (6.5–8.7)
SMBG application, n (%)	36 (12.4)
Duration of T2DM (year), median (IQR)	12 (5–19)
eGFR (mL/min/1.73 m^2^), median (IQR)	64.86 (46.8–86.2)
Chronic kidney disease	
Stage I, n (%)	68 (23.4)
Stage II, n (%)	98 (33.7)
Stage IIIA, n (%)	58 (19.9)
Stage IIIB, n (%)	39 (13.4)
Stage IV, n (%)	20 (6.9)
Stage V, n (%)	8 (2.7)
T2DM therapy	
SU, n (%)	109 (37.5)
Gliclazide, n (%)	24 (22.0)
Gliquidon, n (%)	46 (42.2)
Glimepiride, n (%)	41 (37.7)
Insulin	177 (60.8)
Basal only, n (%)	29 (16.4)
Bolus only, n (%)	6 (3.4)
Mix, n (%)	31 (17.5)
Basal-bolus, n (%)	111 (62.7)
Combination with SU and insulin, n (%)	27 (9.3)
Other, n (%)	31 (10.7)
Severe hypoglycemia, n (%)	74 (25.4)

*SD* standard deviation, *IQR* interquartile range, *SMBG* self-monitoring blood glucose, *T2DM* type-2 diabetes mellitus, *eGFR* estimated glomerular filtration rate, *SU* sulfonylurea.

The proportion of severe hypoglycemia was 25.4%. Bivariate analysis shows HbA1c less than 7%, subjects’ understanding of hypoglycemia symptoms, duration of T2DM more than 10 years, history of severe hypoglycemia, eGFR less than 60 mL/min/1.73 m^2^, use of sulfonylurea, and insulin use were associated with severe hypoglycemia (Table 2). Multivariate analysis shows the history of previous event(s) of severe hypoglycemia (OR 5.864, p ≤ 0.001), eGFR less than 60 mL/min/1.73 m^2^ (OR 1.976, p = 0.028), and insulin use (OR 2.257, p = 0.021) were significantly associated with severe hypoglycemia (Table 3).

**Table 2:** Bivariate analysis of association between independent variables and severe hypoglycemia.

Variable	Severe hypoglycemia		p value
	Yes	No	
Sex, n (%)			
Female	45 (24.6)	138 (75.4)	0.773
Male	29 (26.9)	79 (73.1)	
Age, n (%)			
More than 65	17 (18.1)	77 (81.9)	0.065
Less than 65	57 (28.9)	140 (71.1)	
HbA1c, n (%)			
Less than 7%	20 (18.2)	90 (81.8)	0.038
More than 7%	54 (29.8)	127 (70.2)	
Education level, n (%)			
Less than 9 years	17 (22.1)	60 (77.9)	0.525
More than 9 years	57 (26.6)	157 (73.4)	
Subjects’ understanding of hypoglycemia symptoms, n (%)			
No	26 (17.7)	121 (82.3)	0.003
Yes	48 (33.3)	96 (66.7)	
Duration of T2DM, n (%)			
More than 10 years	48 (31.4)	195 (68.6)	0.021
Less than 10 years	26 (18.8)	112 (81.2)	
History of severe hypoglycemia, n (%)			
Yes	35 (58.3)	25 (41.7)	< 0.001
No	39 (16.9)	192 (83.1)	
SMBG, n (%)			
No	63 (24.7)	192 (75.3)	0.582
Yes	11 (30.6)	25 (69.4)	
Chronic liver disease, n (%)			
Yes	2 (40.0)	3 (60.0)	0.604
No	72 (25.2)	214 (74.8)	
eGFR, n (%)			
Less than 60 (mL/min/1.73 m^2^)	40 (32.0)	85 (68.0)	0.036
More than 60 (mL/min/1.73 m^2^)	34 (20.5)	132 (79.5)	
Sulfonylurea, n (%)			
Yes	19 (17.3)	91 (82.7)	0.019
No	55 (30.4)	126 (69.6)	
Insulin, n (%)			
Yes	60 (45)	117 (66.1)	0.000
No	14 (12.3)	100 (87.7)	

*SMBG* self-monitoring blood glucose, *T2DM* type-2 diabetes mellitus, *eGFR* estimated glomerular filtration rate.

**Table 3:** Multivariate analysis of association between independent variables and severe hypoglycemia.

Variable	OR	p value
Model 1		
Age	0.511	0.061
HbA1c less than 7%	1.009	0.981
Subjects’ understanding of hypoglycaemia symptoms	0.657	0.188
Duration of T2DM	1.259	0.485
History of severe hypoglycaemia	5.346	< 0.001*
eGFR less than 60 mL/min/1.73 m^2^	2.006	0.026*
Sulfonylurea	1.113	0.797
Insulin use	2.134	0.094
Model 2		
Age	0.511	0.061
Subjects’ understanding of hypoglycaemia symptoms	0.657	0.186
Duration of T2DM	1.257	0.476
History of severe hypoglycaemia	2.344	< 0.001*
eGFR less than 60 mL/min/1.73 m^2^	2.008	0.025*
Sulfonylurea	1.114	0.796
Insulin use	2.130	0.088
Model 3		
Age	0.517	0.063
Subjects’ understanding of hypoglycaemia symptoms	0.661	0.190
Duration of T2DM	1.254	0.481
History of severe hypoglycaemia	5.337	< 0.001*
eGFR less than 60 mL/min/1.73 m^2^	2.012	0.025*
Insulin use	1.995	0.058
Model 4		
Age	0.531	0.073
Subjects’ understanding of hypoglycaemia symptoms	0.641	0.155
History of severe hypoglycemia	5.449	< 0.001*
eGFR less than 60 mL/min/1.73 m^2^	2.027	0.023*
Insulin use	2.097	0.038*
Model 5		
Age	0.514	0.058
History of severe hypoglycemia	5.864	< 0.001*
eGFR less than 60 mL/min/1.73 m^2^	1.976	0.028*
Insulin use	2.257	0.021*

*eGFR* estimated glomerular filtration rate, *T2DM* type-2 diabetes mellitus.

*Statistically significant.

Based on the development of the risk calculation model for severe hypoglycemia, the score for the history of severe hypoglycemia, eGFR less than 60 mL/min/1.73 m^2^, and insulin use were 2, 1, and 1, respectively (Table 4).

**Table 4:** Scoring of severe hypoglycemia risk factors.

Variable	B	SE	B/SE	Score
History of severe hypoglycemia	1.769	0.331	5.344	2
eGFR less than 60 mL/min/1.73 m^2^	0.681	0.309	2.203	1
Insulin use	0.814	0.352	2.312	1

*eGFR* estimated glomerular filtration rate.

## Discussion

Our study observed that one-fourth of the subjects experienced severe hypoglycemia. Moreover, eGFR less than 60 mL/min/1.73 m^2^, history of previous severe hypoglycemia events, and insulin use were associated with increased risk of severe hypoglycemia. Using those three factors, we developed a simple hypoglycemia prediction model.

This study observed a high proportion of severe hypoglycemia in T2DM subjects in the Outpatient Endocrinology Clinic of Dr. Cipto Mangunkusumo National General Hospital (25.4%) compared to similar studies in other countries^21–23^. This might be because our hospital is a national referral tertiary-care hospital in Indonesia. Thus, most of the cases were complex. Compared to other studies, our study subjects had a longer duration of T2DM, a higher proportion of insulin use, and a higher proportion of CKD, which contribute to the higher incidence of severe hypoglycemia^21–24^.

This study found eGFR less than 60 mL/min/1.73 m^2^ associated with severe hypoglycemia (OR 1.976, p = 0.028). CKD may increase the risk of hypoglycemia, and insulin resistance is increasingly more frequent at progressively lower Glomerular Filtration Rate (GFR) levels^25^. The Action to Control Cardiovascular Risk in Diabetes (ACCORD) trial study reported that severe hypoglycemia more commonly appears in participants with a lower eGFR^26^. Moreover, 16.2% of patients in intensive therapy had an excess mortality rate^25^. Alsahli et al.^27^ found that eGFR less than 60 mL/min was an independent risk factor for hypoglycemia in 40% of people with diabetes, thus increasing the risk of cardiovascular disease and death. The current study also found that hypoglycemia is higher in patients with CKD than those without CKD due to the decreasing insulin degradation in peripheral tissue^28^. Moreover, CKD patients are at a greater risk of hypoglycemia in cases involving decreased insulin degradation in peripheral tissue^29^.

Our investigation showed association between history of severe hypoglycemia and increased risk of experiencing a subsequent event (OR 5.864, p ≤ 0.001). The finding was consistent with other studies^7,12,21,24,30^. Although the presence of a history of severe hypoglycemia is known to have an association with the next event of severe hypoglycemia, the onset of past hypoglycemia events that could predict the next event of severe hypoglycemia has not been specifically studied. Meta-analysis showed that among studies included, the onset of history of severe hypoglycemia varied, ranging from 1 month to 22 years^30,31^. Blood glucose levels will decrease in subjects with a history of hypoglycemia. A study by Iwase et al.^32^ showed that a history of severe hypoglycemia was a risk factor for developing severe hypoglycemia in type 2 diabetes (HR = 2.38). It will trigger the counter-regulatory mechanism so that hypoglycemia can be more severe and unrealized^33^. Patients who have experienced hypoglycemia can cause the sensitivity of receptors to blood sugar levels to be less sensitive. The signs and symptoms of hypoglycemia are not immediately seen until blood glucose levels are lower than the usual limit for people experiencing hypoglycemia. A history of hypoglycemia indicates hypoglycemia unawareness^34^. The presence of hypoglycemia unawareness increases the risk of severe hypoglycemia (17-fold in T2DM)^35^. Several factors influence the emergence of unawareness hypoglycemia, including comorbidities in the patient, advanced age, and length of suffering from diabetes and type 1 diabetes^36^. Limitations of blood sugar levels that cause severe hypoglycemia are still controversial, but the most important thing is a decrease in consciousness that causes patients to be unable to help themselves from hypoglycemia^37^. In some literature, severe hypoglycemia is caused by SU class of drugs such as glibenclamide. In this study, our SU patients found 37.5% but there was no significant association between SU and severe hypoglycemia. Probably may be due to the dominance of the use of more insulin or SU in combination with insulin.

Furthermore, insulin is well known to be risk factors for severe hypoglycemia^8,9,13^. Rudijanto et al.^5^ showed that 75.1% of T2DM subjects with insulin had hypoglycemia (mild-moderate and severe) within 4 weeks of monitoring. A meta-analysis regarding insulin use showed that the incidence rate of severe hypoglycemia for insulin users in the T2DM population was 23 per patient year^38^. This study showed a significant association between insulin use and severe hypoglycemia (OR 2.257, p = 0.021). One of the reasons for the high proportion of severe hypoglycemia in this study was that most of the subjects were already taking insulin (60,8%), which was 62.7% of them used basal-bolus.

Our multivariate analysis showed eGFR less than 60 mL/min/1.73 m^2^, history of previous event(s) of severe hypoglycemia, and insulin use were associated with increased severe hypoglycemia risk. Based on the scoring we made, a history of severe hypoglycemia has a score of 2, while the scores for using insulin and having an eGFR less than 60 mL/min/1.73 m^2^ are both 1. The range of scores in the scoring system model is a risk factor for the occurrence of severe hypoglycemia which is between 0 and 4. A score of 0 is given if the subject has no risk factors. The higher subject's score, the higher possibility of severe hypoglycemia. For clinical use, we believe that our prediction model might be helpful for clinicians in determining the intensity or adjusting the dose of glycemic management for T2DM subjects, where a higher score may be a consideration for clinicians to provide more intensive glycemic therapy.

However, our study has several limitations, including; (1) study design that leads to potential of recall bias; (2) some variables that are not included as independent variable such as autonomic neuropathy since the diagnosis requires specialized tools, which are not currently used as diagnostic guidelines outside of exceptional cases in our hospital.

## Conclusions

One-fourth of T2DM subjects experienced at least one episode of severe hypoglycemia in a one-year follow-up. Moreover, eGFR less than 60 mL/min/1.73 m^2^, history of severe hypoglycemia, and insulin use were associated with severe hypoglycemia in an outpatient setting of a tertiary care hospital in Indonesia. We managed to develop a simpler severe hypoglycemia prediction model. However, future longitudinal studies are needed to obtain data regarding time-to-event of hypoglycemia.

## Data Availability

The datasets used and/or analyzed during the current study are available from the corresponding author on reasonable request.
